# Three-Dimensional Structure of the Trypanosome Flagellum Suggests that the Paraflagellar Rod Functions as a Biomechanical Spring

**DOI:** 10.1371/journal.pone.0025700

**Published:** 2012-01-03

**Authors:** Louise C. Hughes, Katherine S. Ralston, Kent L. Hill, Z. Hong Zhou

**Affiliations:** 1 Department of Microbiology, Immunology and Molecular Genetics, University of California, Los Angeles, Los Angeles, California, United States of America; 2 California NanoSystems Institute, University of California Los Angeles, Los Angeles, California, United States of America; 3 Molecular Biology Institute, University of California Los Angeles, Los Angeles, California, United States of America; Dalhousie University, Canada

## Abstract

Flagellum motility is critical for normal human development and for transmission of pathogenic protozoa that cause tremendous human suffering worldwide. Biophysical principles underlying motility of eukaryotic flagella are conserved from protists to vertebrates. However, individual cells exhibit diverse waveforms that depend on cell-specific elaborations on basic flagellum architecture. *Trypanosoma brucei* is a uniflagellated protozoan parasite that causes African sleeping sickness. The *T. brucei* flagellum is comprised of a 9+2 axoneme and an extra-axonemal paraflagellar rod (PFR), but the three-dimensional (3D) arrangement of the underlying structural units is poorly defined. Here, we use dual-axis electron tomography to determine the 3D architecture of the *T. brucei* flagellum. We define the *T. brucei* axonemal repeating unit. We observe direct connections between the PFR and axonemal dyneins, suggesting a mechanism by which mechanochemical signals may be transmitted from the PFR to axonemal dyneins. We find that the PFR itself is comprised of overlapping laths organized into distinct zones that are connected through twisting elements at the zonal interfaces. The overall structure has an underlying 57nm repeating unit. Biomechanical properties inferred from PFR structure lead us to propose that the PFR functions as a biomechanical spring that may store and transmit energy derived from axonemal beating. These findings provide insight into the structural foundations that underlie the distinctive flagellar waveform that is a hallmark of *T. brucei* cell motility.

## Introduction

The eukaryotic flagellum (synonymous with motile cilium) is a biological machine that drives fluid movement across epithelial surfaces and propulsion of single cells. Biophysical principles underlying flagellar motility, namely dynein-dependent sliding and bending of adjacent microtubules in the axoneme, are universally conserved, as are the basic arrangements of axonemal sub-structures [1], [2], [3]. Flagellum motility is critical for normal human development and physiology, defects in motile and non-motile cilia cause a broad spectrum of human diseases, collectively referred to as “ciliopathies” [4], [5]. Clinical manifestations of defective cilium motility include infertility, respiratory malfunction and left-right axis defects [4], [5]. Flagella are also required for the motility of several important human pathogens that infect approximately 0.5 billion people worldwide [6], [7]. Movement of these microbial pathogens through heterogeneous media, e.g. host blood and tissues, imposes particular demands on cell motility mechanisms [7], [8] and their flagella exhibit characteristic, cell-specific beat forms [5], [9], [10]. Because the fundamental principles of axonemal motility are conserved, differences in beat form from one cell to another depend on cell-specific elaborations on flagellum architecture. Understanding structural foundations of flagellar motility has the potential to impact efforts to control and treat heritable and infectious disease in humans.

*Trypanosoma brucei* is a uniflagellated protozoan parasite that causes significant human mortality and limits economic development across sub-Saharan Africa. Without treatment, the human disease, known as African sleeping sickness, is fatal [11]. Flagellum-dependent trypanosome motility is central to transmission and disease pathogenesis [7], [8], [12]. Motility of the flagellum drives cell propulsion in the vertebrate host and insect vector, influences cell division, and has been implicated in immune evasion in the mammalian bloodstream [13], [14], [15], [16], [17]. Besides its canonical role in motility, the *T. brucei* flagellum functions in cell morphogenesis and host-parasite interactions [18], [19], [20].

**Figure 1:**
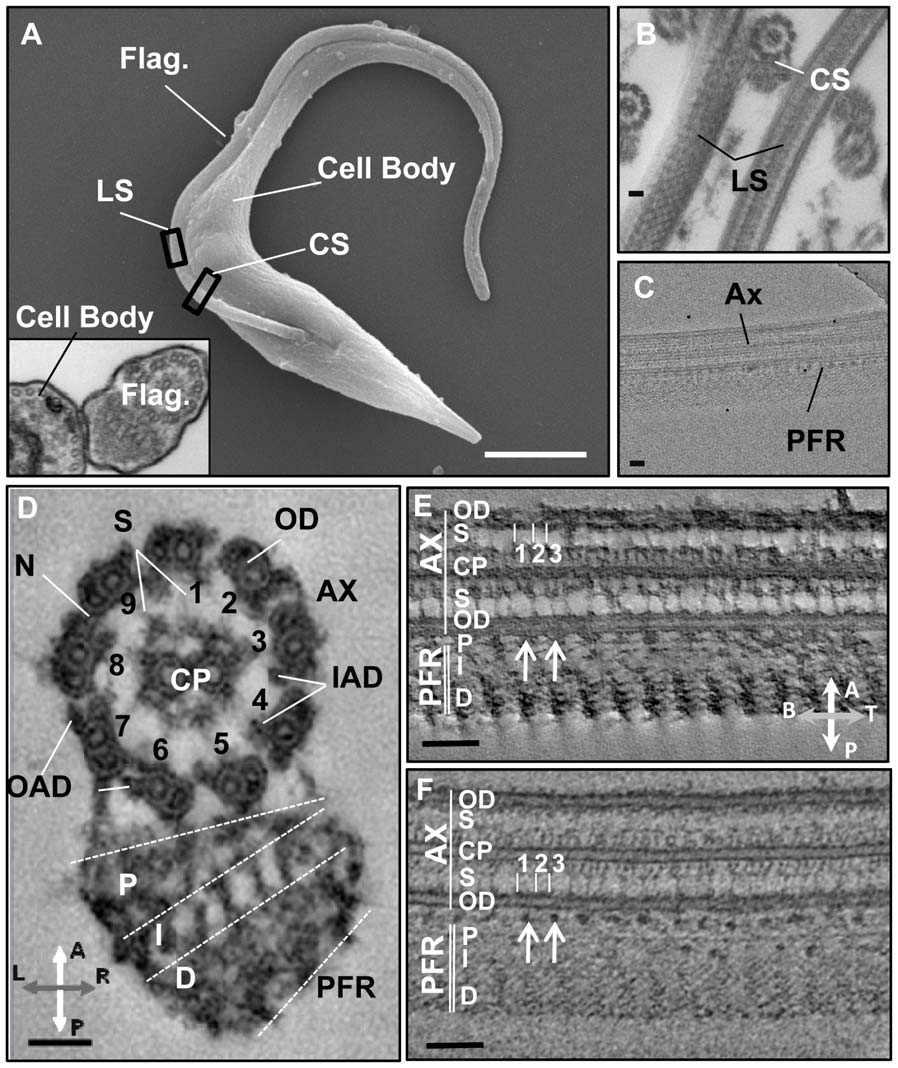
Stained and frozen-hydrated flagellum skeletons provide consistent structural information. (A). Scanning electron microscopy image of a *T. brucei* procyclic cell. Inset shows cross-section TEM image. (B) Resin-embedded samples exhibit a range of orientations, cross-section (CS) and longitudinal-section (LS) are shown. (C) Frozen-hydrated samples in LS orientation show the axoneme (AX) and paraflagellar rod (PFR) side by side. (D) CS view from tomogram of stained samples (slice thickness≈50nm). Outer doublet microtubules (OD), radial spokes (S), outer arm dyneins (OAD), inner arm dyneins (IAD), central pair microtubules (CP) and nexin links (N) are indicated. Ax-PFR Connections are visible. The proximal (P), intermediate (I) and distal (D) PFR zones are indicated on both CS (D) and LS (E–F) views. LS views from tomograms of stained (E) and frozen (F) samples show repeating structures along the flagellum (≈3nm thick). Compasses (E,F) show sample orientation with respect to axoneme (A), PFR (P), base (B), tip (T) and left (L) and right (R) sides (viewed from flagellum base to tip). Scale bars 1 µm (A), 100nm (B–C, E–F) and 50nm (D).

The *T. brucei* flagellum exhibits an unusual, bihelical beat, in which helical waves of alternating handedness propagate from flagellum tip to base and drive cell movement with the flagellum tip leading [10]. One of the most striking features of T. brucei cell architecture is lateral connection of the flagellum to the cell [20] (Fig. 1A), which allows the flagellar waveform to be directly transmitted to the cell body, causing the cell to move in a distinctive auger-like fashion that provided the basis for naming of the genus [21]. There are several additional distinctive features of *T. brucei* flagellum architecture that impact flagellum waveform. Most notably the *T. brucei* flagellum contains a structure known as the paraflagellar rod (PFR) that is attached along the length of the axoneme [22], [23] and is required for trypanosome motility[24], though its precise function is unclear. The PFR is unique to trypanosomes and a few related organisms, but extra-axonemal structures are frequently observed in a number of different organisms, such as the outer dense fibers of human sperm flagella [25]. Despite the central importance of the flagellum and flagellar motility to *T. brucei* biology and disease, the three-dimensional (3D) structural organization of the axoneme and PFR, particularly their relationships to one another, has not been defined.

Studies of flagellar ultrastructure in trypanosomes have traditionally used TEM techniques and freeze-fracture techniques that rely on interpreting 3D structure from 2D projection images [22], [23], [26], [27], [28], [29], [30]. 3D models put forward in these studies propose a lattice-like arrangement in the proximal and distal zones, with an alternate arrangement of filaments in the intermediate zone. In the present study we take advantage of the emerging technology of electron tomography (ET) [31], [32] to define the 3D organization of the axoneme and PFR in *T. brucei*. Our studies provide the first quantitative description of the *T. brucei* axonemal repeating unit and provide insight into flagellum assembly and function. Given the importance of the flagellum to *T. brucei* biology and infection, our findings are relevant to understanding the mechanism of disease pathogenesis and development of novel therapeutics.

## Results

We collected and analyzed a total of 439 tomographic datasets containing flagella (Video S1) from both frozen, hydrated flagellar skeletons (217 tilt series) (Video S2) and samples prepared using traditional chemical fixation and embedding techniques (222 tilt series) (Fig. 1B–F and Videos S1, S2 and S3). Frozen, hydrated samples offer imaging of native structures in the absence of stain, while stained samples offer high contrast and specimen stability, permitting visualization of fine heterogeneous structural features (Figure S1). The presence of PFR posed a limitation for frozen-hydrated samples, as the PFR and axoneme lie side by side on the grid, restricting possible specimen orientations (Fig 1C). Chemical fixation and resin embedding, on the other hand, provided multiple flagellum orientations in each section (Fig. 1B). Multiple orientations in stained samples facilitated compensation for missing wedge artifacts that otherwise reduce resolution in the z plane of any individual tomogram. The missing wedge was further minimized by employing dual-axis tomography.

**Figure 2:**
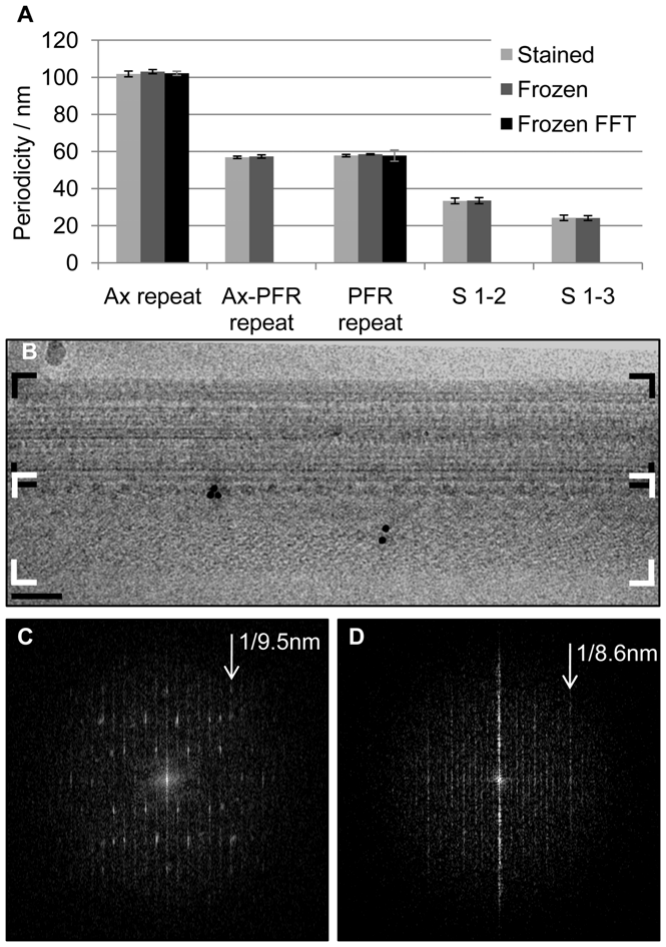
Quantitative analysis of data reveals axoneme and spoke periodicity. (A) Quantitative analysis of the axonemal repeat periodicity (Ax repeat, stained n = 77, frozen n = 45, FFT n = 5), PFR repeat (PFR-repeat, stained n = 299, frozen n = 34, FFT = 6), periodicity of axoneme-PFR interface densities (Ax-PFR repeat, stained n = 102, frozen n = 82) and distance between spoke heads (S 1–2, stained n = 116, frozen = 48), (S 2–3, stained n = 112, frozen = 12),. Error bars show standard deviation. (B) TEM image of a frozen flagellar skeleton. Boxed regions of the axoneme (Black corner markers) and PFR (White corner markers) were used to produce the Fourier transform power spectrums shown in C and D respectively. The 9.5nm and 8.58nm (C and D respectively) positions are indicated.

Ultrastructure was well-preserved in all specimens, as indicated by the presence of all major axonemal structures, including the 9+2 axoneme, outer and inner arm dyneins, nexin links, radial spokes and central pair complex; together with axoneme-PFR connectors and the proximal, intermediate and distal zones of the PFR (Fig. 1D–F). Asymmetry of the axoneme-PFR interface and orientation of dynein arms allowed unambiguous identification of outer doublets, which are numbered 1–9 according to convention [33], [34] (Fig. 1D). No significant differences in the fundamental dimensions of major structural landmarks were observed between stained and frozen-hydrated samples (Fig. 2), indicating that both provide faithful preservation of native flagellar substructures.

### The repeating unit of *T. brucei* axoneme and PFR

**Figure 3:**
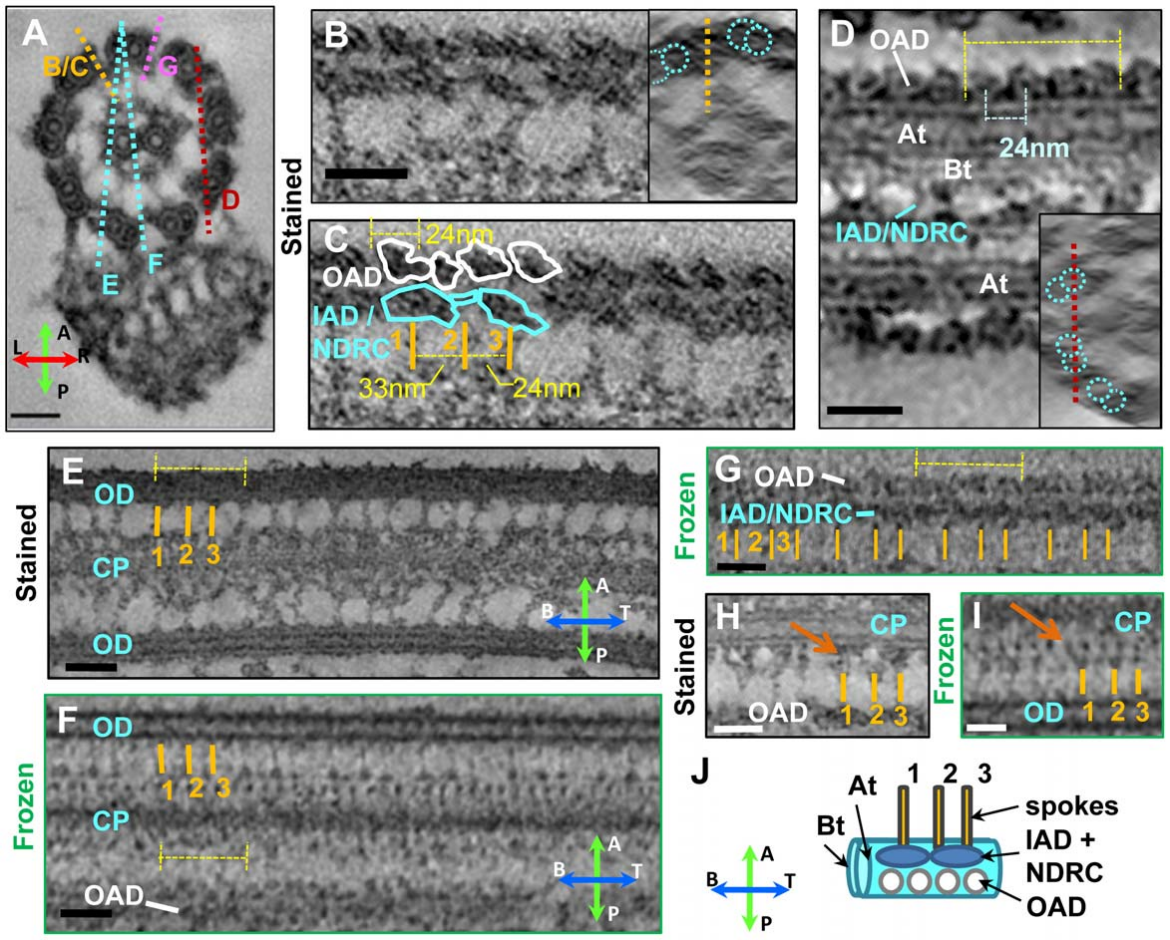
The paraflagellar rod repeat unit is synchronous with the axoneme repeat unit. Panels A to H show digital slices of tomograms. (A) Cross-section (CS) slice of a stained flagellum skeleton (also Fig. 1E) shows positions of slices in panels B–G. (B, C) Slice through radial spokes (1–3), IAD and OAD. Raw data is shown in panel B, the same image with annotation is shown in C. Panel B inset shows a CS view of the same tomogram, with outer doublet microtubules (blue) position of slice (orange line). (D) Slice through outer doublet microtubules. Inset shows a CS view of the same tomogram, for slice position reference. A (At) and B (Bt) tubules of the outer doublet are shown, as are OAD motor domains and the IAD/NDRC complex. (E, F) Digital slices of stained (E) and frozen (F) samples bisect the central pair microtubules (CP), outer doublet microtubules (OD) or outer arm dyneins (OAD). Axoneme repeat is indicated (yellow lines). (G) Slice of frozen sample showing the OAD and IAD/NDRC with respect to the triplet spoke repeat along the axonemes. Slice of stained (H) and frozen (I) samples show radial spokes connected to central pair microtubules (CP) via filaments (red arrows). (J) Diagram summarizing the axoneme repeat. Abbreviations are as indicated in Fig. 1. Compasses in panels A and F show sample orientation as described in Fig. 1. Digital slice thickness is 50 nm (A) or 3 nm (B–H). Scale bars are 50nm.

Composition and arrangement of the trypanosome axonemal repeating unit has not previously been defined. Digital slices of tomograms were used to define the trypanosome axonemal repeat unit and determine architectural spacing of core substructures (Fig. 3). These numbers were independently confirmed by calculating the spatial frequencies in the horizontal direction (along the long axis of the flagellum) of the first order spots, which are visible in the Fourier transforms (FFT) of PFR and axoneme images (Fig. 2C–D). A group of three radial spokes was repeated approximately every 100 nm along axoneme (Fig. 2) and defined the repeat unit of the axoneme. Spoke triplets were observed along every outer doublet connected to the A tubule. The distance between spoke heads 1 and 2, numbering from base to tip of the flagellum, was 33 nm (±1.5nm, n = 164) and the distance between spoke heads 2 and 3 was 24 nm (±1.5nm, n = 154). Each triplet set of spoke heads contacted a network of fibrils projecting from central pair microtubules (Fig 1E–F, 3E–I). Six fibrillar projections were generally observed per triplet group of spoke heads. Spoke heads exhibited a bi-helical arrangement around the central pair apparatus, forming two hemi-helices of alternate handedness (Fig. S2). Outer arm dyneins were clearly resolved and structures resembling ring-shaped motor domains [35] were resolved in some samples (Fig. 3D). Outer arm dyneins were arrayed along the A-tubule with a periodicity of approx. 24nm (±3nm, n = 42), thus giving 4 outer arm dyneins per axoneme repeat. Two large clusters of mass density per repeat were apparent on the inner face of each A tubule, beneath the 4 outer arm dyneins, and overlapped with the sites of spoke attachment. Based on their positions relative to other structures, we interpreted these densities to encompass the inner arm dyneins and nexin-dynein regulatory complex [36].

Longitudinal views of the PFR-axoneme interface revealed mass densities that repeat every 57.2 nm (±1nm, n = 109 stained; n = 82, frozen) along the length of the PFR (Fig. 1E–F, 2). Digital slices that bisected the radial spokes and the PFR showed that there were approximately two of these densities per axonemal repeat (Fig. 1E–F). A similar longitudinal periodicity (57.9 nm±1nm, n = 299, stained; n = 34, frozen; n = 6, FFT) was observed for bands of density running through the PFR distal domain (Fig. 1E–F, 2).

### The paraflagellar rod is directly connected to outer arm dyneins

**Figure 4:**
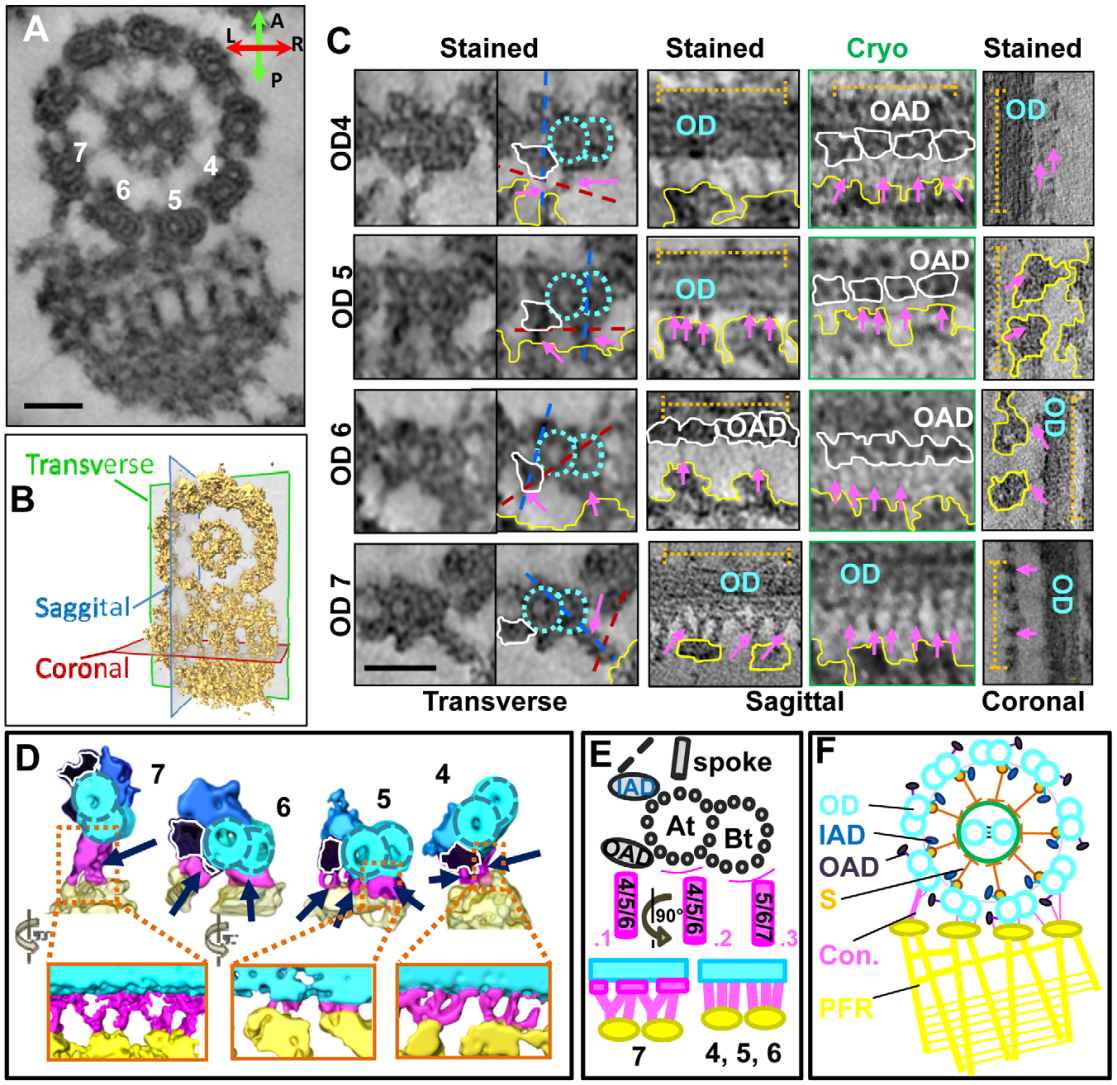
Axoneme-PFR connectors. (A) CS slice shows positions of outer doublets (OD) 4, 5, 6 and 7 adjacent to the PFR. (B) Surface rendering of data (A) with relative orientation of transverse, sagittal and coronal planes. (C) Transverse, sagittal and coronal tomogram slices at the PFR interface with OD4 -7. Two identical transverse slices, unannotated (left) and annotated (right), are shown. Stained and frozen data are indicated. Ax-PFR connectors (pink arrows) connect the PFR (yellow) to OAD (white), and OD (dashed light blue). Positions of stained sagittal (dashed dark blue) and coronal (dashed red) views are shown. Length of the 96-nm axoneme repeat is shown (dashed orange). (D) Surface rendering of segmented tomograms show transverse (top) and sagittal (bottom) views of OD (light blue), OAD (dark purple), IAD (dark blue), connectors (pink) and PFR (yellow). (E,F) Diagrams summarize arrangement of Ax-PFR connectors. (E) Transverse and sagittal detail of OD-PFR interface, with connectors represented in pink, is shown. Connectors (.1-.3) present at a given OD-PFR interface are shown with corresponding OD number (4, 5, 6 or 7). (F) Diagram in transverse view shows the position of each Ax-PFR connector (Con.). Abbreviations are as defined in Fig. 1. Digital slice thickness is 50 nm (A) or 3 nm (C). Scale bars are 50nm.

Electron dense filaments provide a physical connection between the PFR and axoneme in the region between outer doublets (OD) 4, 5, 6 and 7 [26], [27]. We found that the axoneme-PFR connectors (Fig. 4) exhibited a defined radial organization, as revealed in transverse views, as well as a longitudinal repeating pattern, as revealed in sagittal and coronal views. We provide here a general description that encompasses the main structural elements (summarized in Fig. 4E, F).

Transverse views of the OD-PFR interface showed a radial organization of connectors to OD4, 5 and 6. In most cases one connector appeared to be (4.1, 5.1 and 6.1) attached directly to the outer arm dynein and the others attached to the A tubule, near the A tubule-B tubule (At-Bt) interface (4.2, 5.2, 6.2), and the B tubule (5.3 and 6.3) (Fig. 4C–F). Because all three connectors were not co-planar they were not always visible at the same time in the thin (3 nm) transverse slices. The OD7-PFR interface was uniquely characterized by a single thick connector (7.3) attached to the B tubule.

Axoneme-PFR connectors were examined in further detail using sagittal and coronal views of digital slices from tomograms of stained samples and sagittal view of digital slices from frozen samples. (Fig. 4C, D). These analyses confirmed the sites of connector attachment to outer arm dyneins and outer doublet microtubules. Additionally, these views revealed complexity of axoneme-PFR connections in the longitudinal axis, showing that several filamentous connectors emanated from each of the mass densities, described above, that occur at 57.2nm intervals along the axoneme-PFR interface (Fig. 4C). Connectors varied in length (7–28nm), depending on the distance between the axoneme and PFR. In longitudinal views, the OD7-PFR interface was again distinguished from the other connections and was comprised of a triplet of connectors. Two of the OD7-PFR connectors formed a Y-shaped structure, while the third remained singular (Fig. 4C–E), which correlates with connectors described previously [26], [27], [37].

### The paraflagellar rod is composed of overlapping laths that are contiguous through the proximal, intermediate and distal domains

**Figure 5:**
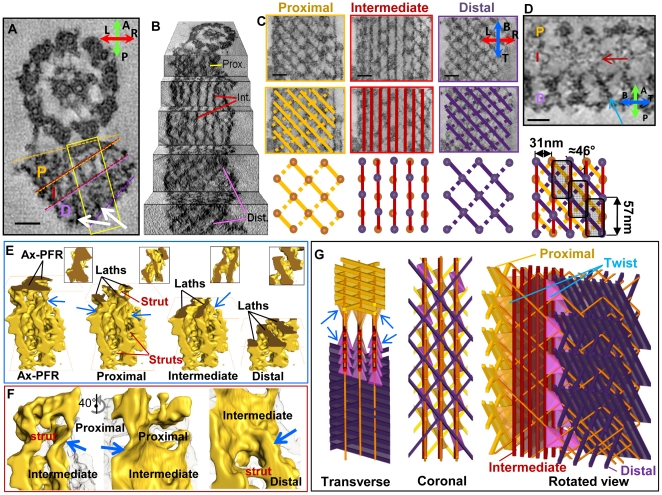
Structure of the PFR. (**A**) CS slice showing transverse views of the flagellum skeleton (slice thickness 1nm) showing the proximal (P), intermediate (I) and distal (D) PFR zones. Densities extending across the PFR can be seen (white arrows). Yellow box indicates position of data in E–F. (B) Perspective view of tomogram data, with P, I and D zones indicated. (C) Coronal slices of Ax-PFR interface and PFR zones (as indicated) show raw data (top), data with laths and struts highlighted by solid and dashed lines, respectively (middle) and diagrams (bottom). Circles indicate lattice intersection points. (D) Top: Sagittal slice (3nm) through each of the zones showing parallel filaments in the intermediate zone (red arrow) and orthogonal filaments extending from the proximal through distal zones (blue arrow). Bottom: composite diagram showing PFR zones in projection with a LS periodicity of 48 nm and a structural repeat unit (shaded rectangles). (E) Surface rendering of region shown in A, cut through the Ax-PFR interface, P, I and D zones, as indicated. Insets show face-on views of each cut surface. (F) Enlargement of structure shown in E. Left: Same orientation as in E, shows twisting of structure (blue arrow) at the P-I zone interface and struts (red text). Middle: Same region shown in left rotated 40° to show wall-like intermediate lath. Right: same orientation as middle shows twisting of structure at I-D interface (blue arrow). D zone struts are indicated. (G) 3D graphical diagram of PFR orientated to reflect transverse (tilted slightly to reflect sample orientation in E) and coronal views and a rotated view showing the 3D construction. Zones are colored as shown in C. Twisting elements are indicated (blue arrows and “twist” label) Compasses in panels A, C and D show sample orientation, as described in Fig. 1. See also supporting Videos V3, 4 and 5. Scale bars are 50nm.

Thin (1nm) transverse digital slices (Fig. 5A) showed that lines of density that are hallmarks of the intermediate zone in thicker (70 nm) slices (Fig. 1D, Fig. S3A) were segments of longer lines extending from the proximal through the distal zone. A series of 1-nm thick slices encompassing a total sample thickness of 20 nm showed that continuity of each line was generally retained through the Z axis, but that the X,Y position of the proximal and distal segments varied considerably from one Z-section to the next (Fig. S4 and Video S3). Perspective views of tomograms (Fig. 5B) allowed us to simultaneously visualize transverse and coronal views of each PFR zone. This analysis showed that linear densities observed in transverse planes are different views of densities observed in coronal planes (Fig 5C).

Coronal digital slices through 3 nm of sample thickness revealed distinctive patterns of density in the proximal, intermediate and distal zones (Fig 5C). Slices through the proximal zone gave a lattice-like appearance, with two intersecting arrays of parallel lines, oriented at approximately −46° and +46° degrees relative to the flagellum's long axis. The −46° lines were slightly more distinct. Lines within each array were separated by 40 to 50nm and intersected the other array at 57 nm intervals along the flagellum's long axis (Fig. 5B, C). Digital slices through the intermediate zone showed parallel linear densities, spaced 31nm (±1nm, n = 134) apart, running along the flagellum's long axis. Occasionally, amorphous densities between lines were observed. Each linear density in the intermediate zone was aligned with intersection points of the proximal zone lattice. Digital slices through the distal zone gave an appearance similar to that seen in the proximal zone, but with lattice intersection points shifted 28–29 nm along the flagellum's long axis. A single TEM image encompassing the entire 200-nm thick sample gave a composite view that correlated with the overlap of individual digital slices obtained from tomograms (Fig. S5).

Perspective views of tomograms (Fig. 5B) indicated that diagonal lines of density in the proximal and distal zones were derived from twisting segments of the straight lines in the intermediate zone. This result supports the view from transverse digital slabs that each linear density prominent in the intermediate zone is part of a continuous line of density extending from the proximal through the distal zone. Thin (3nm) sagittal views cutting through all PFR zones revealed stacked filaments parallel to the flagellum's long axis (Fig. 5D, red arrow) and obliquely orientated filaments extending from the proximal through the distal zone (Fig. 5D, blue arrow).

Surface renderings of segmented tomogram volumes provided 3D views of PFR structure, including conformational changes that mark the interface between PFR zones (Fig. 5E–F and Video S4). The proximal zone lattice consisted of two distinct structural elements. The dominant elements, corresponding to the −46° lines of the lattice, were wall-like structures, which we term proximal laths. Adjacent laths were connected by rod-like elements, which we term struts, that correspond to the +46° lines of the lattice. The intermediate zone was also comprised of parallel wall-like structures, termed intermediate laths, which run the length of the flagellum. Proximal and intermediate laths were connected by twisting structural elements at the interface between zones. Similar twisting motifs formed the intermediate-distal zone interface. The distal lattice was comprised of the same structural elements observed in the proximal lattice, namely laths corresponding to the −46° diagonal array, that were interconnected by struts, corresponding to the +46° array. The twisting motifs at the interface between zones, were spaced at 57 nm intervals, with a 28–29-nm offset between the proximal-intermediate and intermediate-distal interface. The combined 3D imaging data indicated a PFR structure composed of a repeating unit that has a longitudinal periodicity of 57-nm and a lateral periodicity of 31 nm (Fig. 5C). Laterally adjacent repeat units were offset by 28–29 nm along the flagellum's long axis.

## Discussion

We define the axoneme and PFR repeat units of the *T. brucei* flagellum and identify connections from the PFR to dynein motors that drive axonemal motility using dual-axis ET and cryo ET. Robustness of the data is evidenced by identical dimensions of major structural landmarks in frozen-hydrated samples and stained, embedded samples. Our findings provide a structural foundation for considering molecular mechanisms of PFR assembly and flagellar motility in trypanosomes.

The number of spokes per axoneme repeat can be either be two, as in *Chlamydomonas* [38], or three, as in mammalian sperm and sea urchins [39], [40]. *T. brucei* has three spokes per repeat (Fig. 1 and 3) and spoke spacing (Fig. 2) agrees well with published dimensions for spoke triplets in other organisms [39], [40], [41]. *T. brucei* spoke heads exhibit a bi-helical arrangement around the central pair apparatus, with two hemi-helices of alternate handedness (Figure S2). This arrangement is similar to that reported for sea urchin sperm [39] and different from the uniform helical arrangement of spoke heads in *Chlamydomonas* and *Tetrahymena* axonemes [39]. The bi-helical arrangement of spoke heads likely imposes structural constraints on the central pair apparatus and might explain the restricted range of central pair microtubule rotation relative to outer doublet microtubules in *T. brucei* and sea urchins [13], [17], [42], [43].

We observed structural diversity among filamentous connectors that link the axoneme to the PFR. Axonemal dynein motors on OD4, 5 and 6 are directly connected to the PFR. To our knowledge, this link and details of the OD4-6 connectors have not been described previously. Morphology of the OD7 connectors reported here indicate they correspond to the axoneme-PFR connectors described in freeze-fracture and TEM studies [26], . PFR-dynein connectors have not been described previously, but are observed upon careful re-examination of published TEM data, for example Figure 4a of [26] and Figure 5 of [44]. The PFR imposes structural restrictions on axonemal beating [24], [45] and is a scaffold for assembly of enzymatic and regulatory activities [46], [47], [48]. These observations have fuelled the hypothesis that the PFR plays an active role in directing axonemal motility [49], although the mechanisms are unclear. We propose that the physical connections between the PFR and outer arm dyneins provide a mechanism by which chemical and/or mechanical regulatory signals originating in the PFR can be directly transmitted to the axonemal dynein motors. Loss of outer arm dyneins causes backward cell locomotion and reversal of the tip-to-base waveform that is a hallmark of trypanosome flagellar motility [13], [50], [51]. Thus, PFR connection to outer arm motors may have particular relevance to the distinctive flagellar beating of trypanosomatids.

The PFR is a distinctive feature of the kinetoplastid flagellum, is required for normal flagellar motility and is essential for parasite viability [14], [24], [52], raising interest in the PFR as a potential drug target [49]. However, despite being described almost fifty years ago [22], PFR structure has remained enigmatic. Here we describe a PFR architecture in which individual structural elements of each PFR zone are interconnected to form a single superstructure. In the intermediate zone, parallel wall-like laths run the length of the flagellum. These laths are connected by twisting structural elements to obliquely oriented laths in the proximal and distal zones, where rod-like struts connect adjacent laths, giving rise to the lattice-like appearance of coronal sections.

The specific architecture described here was revealed using 3D imaging and is, to our knowledge, not described elsewhere. Previous 3D models for PFR structure are based on serial section TEM and freeze-fracture approaches [26], [27], [30], [53]. The most relevant comparisons are with the pioneering works of Fuge [27] on *T. brucei* and Farina [26] on *Herpetomonas* and *Phytomonas.* Recent 2D freeze fracture studies [30] build on earlier work by comparing bent and straight fragments of the structure. Models put forward in these earlier studies [26], [27] have in common a network of thin (∼7 nm) and thick (∼25 nm) filaments (“bands of density” in Fuge) organized into a lattice-like arrangement in the proximal and distal zones, with an alternate arrangement of filaments in the intermediate zone. The salient difference between the earlier models is the organization of filaments in the intermediate zone. Fuge proposes that “longitudinal filaments” in the intermediate zone run parallel to the flagellum's long axis and are stacked on top of one another in the sagittal plane, *i.e.*, from proximal to distal. In the models of Farina [26] and Rocha [30] intermediate zone filaments are angled relative to the coronal plane, extending from the proximal to distal zone. Both sets of filaments are observed in our raw tomogram analyses (Fig. 5D). We thus propose that intermediate zone laths (Fig. 5E, F) correspond to closely-stacked longitudinal filaments of the Fuge model, enmeshed with angled filaments of the Farina model. Proximal and distal zone laths correspond to stacks of thin (7 nm) filaments observed by Fuge and Farina. Rod-like struts that connect adjacent laths in the proximal and distal zones of our model correspond to structures reported as “oblique bands of density” [27], or “thick” (∼25 nm) filaments [26]. Twisting structural elements at the interface between zones are unique features of our model. These features are deep within the structure itself and, therefore, not likely to be revealed in the earlier studies, owing to the inherent limitations of 2D projection images to reveal internal features of 3D objects. A scaled graphical model incorporating our data in the context of earlier models is shown in Figure 4G and Video S5.

Beating of the *T. brucei* flagellum [10] (Video S6) requires that the PFR withstand biomechanical stresses, including shear, torsion, compression and extension. Interconnection of alternating structural motifs, presented by proximal/intermediate/distal PFR zones, each with distinct biomechanical properties, provides an architecture that can withstand extensive structural deformation and recovery. Pivotal to this architecture is the interface between alternating structural motifs, which we observe to be twisted elements connecting laths at the transition between proximal-intermediate and intermediate-distal zones. We suggest that the twisting elements buffer displacements between zones caused by deformation of structure in response to stress. The entire structure can thus accommodate a wide range of movements and further, has the capacity to store potential energy as the flagellum twists. This energy would be released as the structure returns to its relaxed state. Taken as a whole, we propose the PFR superstructure acts as a biomechanical spring, which disperses and transmits energy derived from axonemal beating. Such activity would be particularly useful for facilitating the bihelical waveform that drives *T. brucei* motility [10].

Our model also offers insights into mechanisms of PFR assembly. The twisting interface between zones suggests models for molecular assembly of the PFR. The major PFR protein components are two repetitive proteins, PFR1 and PFR2. The PFR also includes substoichiometric amounts of other proteins containing variations on the repeating amino acid domain in PFR1 and 2 [49]. In our model, lath structures in each zone are composed of stacked filaments. We suggest that these filaments represent polymers of PFR1 and 2, which are located throughout the PFR structure [44]. Incorporation of substoichiometric isoforms of PFR1/2-repeat containing proteins into laths could induce deformation of underlying structure, giving rise to the twists that characterize the interface between zones. This hypothesis could now be tested by employing specific antibodies to determine the location of each isoform.

During the course of review, a separate group reported structural analysis of the T. brucei flagellum using CryoET [54]. In that elegant work, the authors focused on characterizing the repeating unit of the PFR in the straight and bent states. Through subtomographic averaging, based on a 56-nm periodicity, the structures of the distal domain of the PFR in both states were obtained, leading to a “jackscrew” model. The periodicity of the distal domain and portions of the proximal domain in a straight state correlate with our measurements of these regions. This work complements the detailed architectural organization including PFR, axoneme and connectors reported in the current study.

## Materials and Methods

Procyclic 29-13 *T. brucei* parasites [55] were cultivated as described [17]. Asynchronous mid-log-phase cell cultures were collected by centrifugation at 2,000xg and washed three times with 1X PBS. Flagellar skeletons were prepared using a modified version of [56], as detailed in [57]. Briefly, cells were re-suspended to 2×10^8^ cells/ml in PMN buffer, containing 1%NP40 (1% NP40, 10 mM NaPO4 pH 7.4, 150 mM NaCl, 1 mM MgCl2, 25 µg/ml aprotinin, 25 µg/ml leupeptin, and protease inhibitor cocktail). DNase I (Worthington) was added at 0.25 mg/ml to remove mitochondrial DNA. Following a 10 minute incubation at room temperature, samples were incubated on ice for 30 minutes, and flagellar skeletons were collected by centrifugation at 16,000xg for 10 minutes. Flagellar skeleton pellets were subsequently transferred to a fresh tube and washed twice with PMN buffer. This procedure allowed for one-step fractionation to obtain flagellar skeletons and improved sample preservation [57].

### Sample preparation for Transmission electron microscopy

For stained and embedded samples, flagellar skeletons were fixed in 1% glutaraldehyde and 1% paraformaldehyde in 0.1M sodium cacodylate buffer containing 1% tannic acid at pH 7.2, post-fixed in 1% osmium tetroxide with 1.5% potassium ferrocyanide. Samples were dehydrated using an ascending acetone series, followed by infiltration and embedding in Eponate 12 (Ted Pella, Co) as described in [58]. To prepare frozen samples for cryoET, freshly prepared flagellar skeletons in PMN buffer at a concentration of 1×10^10^ cell equivalents/ml were mixed with protein-A gold (10nm) purchased from Cell Microscopy Center of University Medical Center Utrecht at a 70∶1 v/v mixing rate. A 3 µl droplet of the gold-containing flagellar skeleton sample was applied onto 200 mesh Quantifoil 3∶1 µm holey-carbon coated grids, blotted by filter paper, and plunge-frozen in ethane cooled by liquid nitrogen.

### Electron tomography

Electron tomography was performed in an FEI Technai G^2^ TF20, operated at 200kV as previously described [59]. Briefly, the images were recorded using a TVIPS F415MP 16 megapixel CCD camera at an original magnification of 29,000x and 50,000x. Stained sections were imaged using dual-axis tilt tomography with *Batchtomography* software from FEI. Each series was taken with a defocus of −3 µm from −70° to +70° tilt along alpha and beta tilt axes, with 2° increments at the lower tilt angles (range±40°) and 1° increments above +40° and below −40°. Dual axis data sets were achieved by taking single tilt data sets at several points of the grid before manually rotating the grid by 90° and taking the second tilt series at the same locations.

CryoET data sets were taken using low dose imaging protocols using FEI Technai G^2^ TF20, operated at 200kV, and FEI Titan Krios, operated at 300kV using a Gatan energy filter 2002 and 2kx2k CCD camera. Focus and tracking positions were located along the holder tilt axis 5–7 µm away from the exposure area, to prevent exposing the region of interest during focus and tracking. Single tilt data sets from 70° to +70° were taken at a 2° intervals. The defocus used was −5 µm. The dosage of cryo-samples was adjusted so that the total accumulated electron dose for each entire tilt series is between 70–100 e/A^2^.

### Data processing

Images in each tomography tilt series were aligned and combined to generate 3D tomograms using *eTomo* (IMOD, Boulder, CO) [60], [61]. CryoET data were aligned using standard gold bead tracking using a minimum of 5 fiducials. Fiducialless alignment was initially used for stained sample data alignment followed by patch tracking with patches set to 400x400 pixel size. The two tomograms generated for each area of interest were aligned by matching distinctive features in both tomograms and combined also using *eTomo* to create a single final tomogram. To reduce image noise, the tomograms were first filtered using the median function of the clip IMOD program and subsequently low-pass filtered to 40 Å resolution. Z-axis adjustment was made on the stained samples to correct for shrinkage according to IMOD protocols (3Dmod users guide). Segmentation and image processing were conducted using Amira 5.2.0 (Visage Imaging).

Segmentation is based upon distinct characteristics of the data, the density of stain and structural morphology. Regions of interest (ROI) were selected (using the “brush” tool), and data within the ROI displayed using automatic functions (“thresholding” and “smoothing”). Different colors in the volumes were the result of displaying several ROI (“materials”) together. Videos were generated using the demo-maker and movie-maker functions on Amira. Videos of tilt series and tomograms were generated using 3Dmod (IMOD, Boulder, CO) and VideoMach (Version 5.4.8, Gromada) was used to combine images into a video.

Measurements to quantify dimensions in the flagellum were made using the Amira measuring tool of frozen and stained samples. Measurements were confirmed by independently applying Fourier transform to axoneme and PFR images from frozen flagellar skeletons (generated using EMAN, National Center for Macromolecular Imaging, TX). Spatial frequencies were calculated along the horizontal plane (along the long axis of the flagellum) of the power spectrum using the bright spots as markers. Images used for the diffraction data were taken with the TF20 and Titan Krios microscopes and boxed to contain only axoneme or PFR structures (Fig. 2B). All analyses were conducted with relatively straight segments of flagellar skeletons, so as to minimize the influence of sample curvature on arrangement of structural elements. Measurements using Amira were made between edges of structures to capitalize on the high contrast.

## Supporting Information

Figure S1 **Imaging of stained versus frozen-hydrated samples.** (A). Schematic diagram comparing image interpretation for stained and cryo (frozen-hydrated) data. Stain accumulates around and within structures, sharply delineating edges in the final images. There is no stain in the frozen/cryo samples, thus electrons are deflected by the sample itself. (B) Both stained and frozen-hydrated samples are tilted to create a series of projection images. Weighted back-projection of aligned images creates a 3D tomogram. (C) Tomogram volumes can be viewed in x, y and z planes.
(TIF)

Figure S2 **Bihelical arrangement of spoke triplets around the central pair microtubules.** (A) Cross-sectional tomogram slice (≈50nm thick) shows a transverse view of the flagellum skeleton. Color scheme for spoke heads is based on outer doublet number as shown on the left, with outer doublet numbering as shown in figure 1. (B–E) Surface renderings of segmented tomograms show position of spoke heads around the central pair microtubules (light blue). Sample is oriented with flagellum base at left. Only the #1 spoke head from each triplet is shown, colored according to panel A. Each image is a 90° rotation toward the viewer of the previous image (B–E). White lines tracing the shortest distance to the spoke on the adjacent doublet moving 1 to 9, yield two hemi-helices of alternate handedness. Scale bars are 50nm.
(TIF)

Figure S3 **Serial tomogram slices of a frozen flagella skeleton showing the axoneme and PFR repeat.** (A–E) Longitudinal section tomogram slices (≈1nm thick) show the axoneme and PFR repeat. (A) OD7-PFR connectors are in sync with periodic PFR densities. (B) OD6-PFR connectors are attached to periodic PFR densities in the proximal zone (Prox. PFR). Longitudinal fibrils are present in the intermediate (Int. PFR) and distal (Distal PFR) PFR zones. (C) Spoke triplets are arranged along the axoneme between the central pair microtubules and the outer doublets. (C–E) Distinct bands of periodic densities can be observed in the PFR (arrows). Scale bars are 100nm.
(TIF)

Figure S4 **CS slices showing contiguous densities extending from proximal through to distal zones of the PFR.** Transverse views of the flagellum skeleton. Slice thickness is indicated in each panel. The 70-nm thick slice shows the proximal (P), intermediate (I) and distal (D) PFR zones. Parallel linear densities are (white arrow) visible in the intermediate zone. Thin slices (10nm and 1nm) show densities extending across the PFR (white arrows). The x-y position of the proximal and distal segments of these contiguous densities displays variability in sequential thin z-slices. See also Video S2. Scale bar is 50nm.
(TIF)

Figure S5 **TEM image showing the relative positions of laths in each PFR zone.** TEM image (∼200-nm thick) is of the same sample shown in 4C. Colored arrows indicate the position and direction of laths in the proximal (gold), intermediate (red) and distal (purple) zones. Proximal and distal laths are offset from one another by 24nm. Compass shows sample orientation, as described in Fig. 1. Scale bar is 50nm.
(TIF)

Figure S6 **Digital slice from tomograms showing filaments in the PFR intermediate zone.** (A, B) Sagittal tomogram slices (≈3nm thick) from a stained (A) and frozen (B) sample showing the 3 PFR zones, proximal (gold), Intermediate (red) and distal (purple). Filaments are observed in the intermediate zone parallel to the long axis of the flagellum (red arrows) and obliquely orientated, forming a connection from proximal through distal zones (blue arrows). Scale bar is 50nm.
(TIF)

Video S1 **Aligned tilt series of stained flagella skeletons.**
(AVI)

Video S2 **Serial Z slices through a tomogram volume.** The frozen-hydrated flagella skeleton (also shown in supporting figure S3) shows the periodicity of the PFR and axoneme. Connectors between the PFR and outer doublets are also seen.
(AVI)

Video S3 Serial Z slices through a tomogram volume. Cross-section of flagella skeleton, (also shown in Figure 4A) shows variable positions of densities in the PFR running from the proximal zone through the distal zone.
(AVI)

Video S4 **Surface rendering of segmented volume.** Segmented volume (also shown in figure 4E–G) shows twists in structures at the proximal-intermediate and intermediate-distal interfaces. The volume is cut away to reveal lath and strut orientation in each PFR zone.
(AVI)

Video S5 **Three-dimensional graphical model of the PFR.**
(AVI)

Video S6 **Motility of a procyclic-form** ***T. brucei*** **cell.**
(WMV)
