# Supplementary figures and images for: Three-Dimensional Structure of the Trypanosome Flagellum Suggests that the Paraflagellar Rod Functions as a Biomechanical Spring
Source: PLoS One. 2012 Jan 3;7(1):e25700. doi: 10.1371/journal.pone.0025700 (PMC3250385; doi:10.1371/journal.pone.0025700)

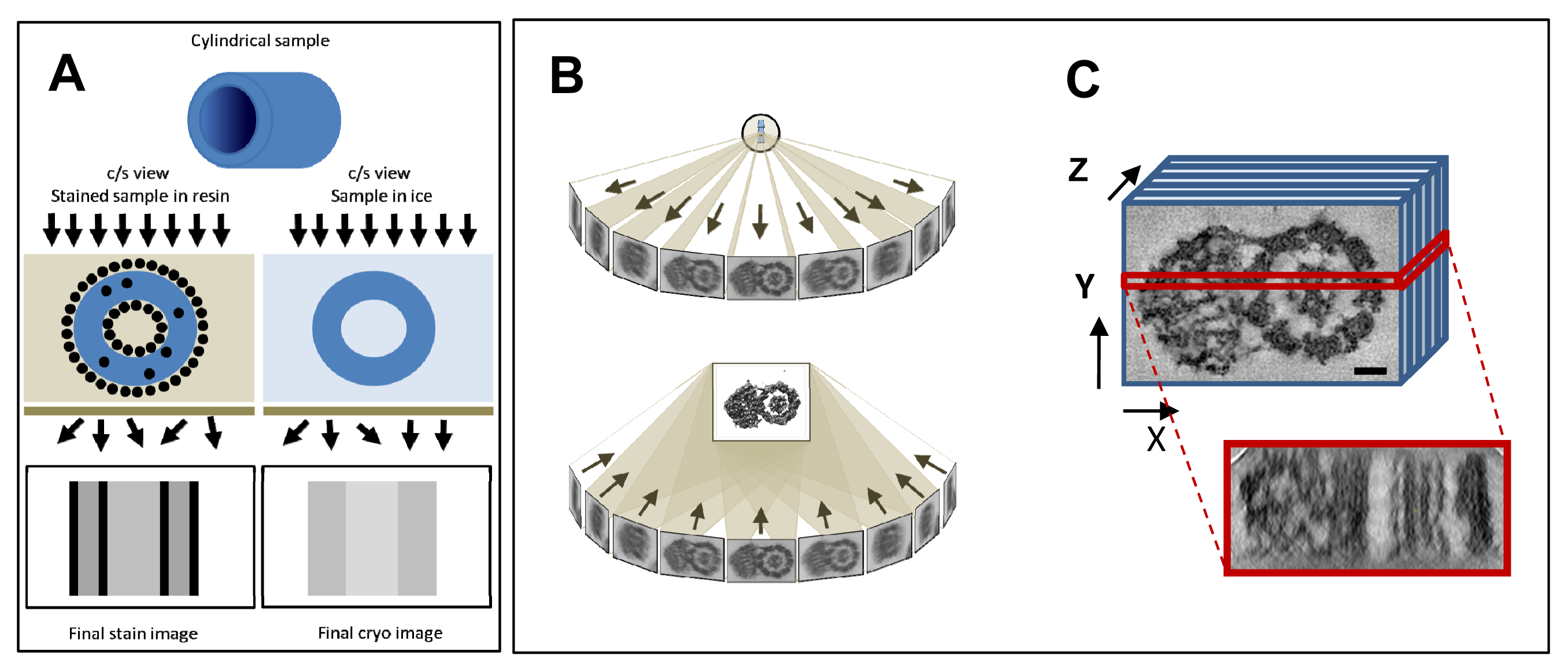

Supplement: Figure S1 — Imaging of stained versus frozen-hydrated samples. (A). Schematic diagram comparing image interpretation for stained and cryo (frozen-hydrated) data. Stain accumulates around and within structures, sharply delineating edges in the final images. There is no stain in the frozen/cryo samples, thus electrons are deflected by the sample itself. (B) Both stained and frozen-hydrated samples are tilted to create a series of projection images. Weighted back-projection of aligned images creates a 3D tomogram. (C) Tomogram volumes can be viewed in x, y and z planes. (TIF) [file pone.0025700.s001.tif]

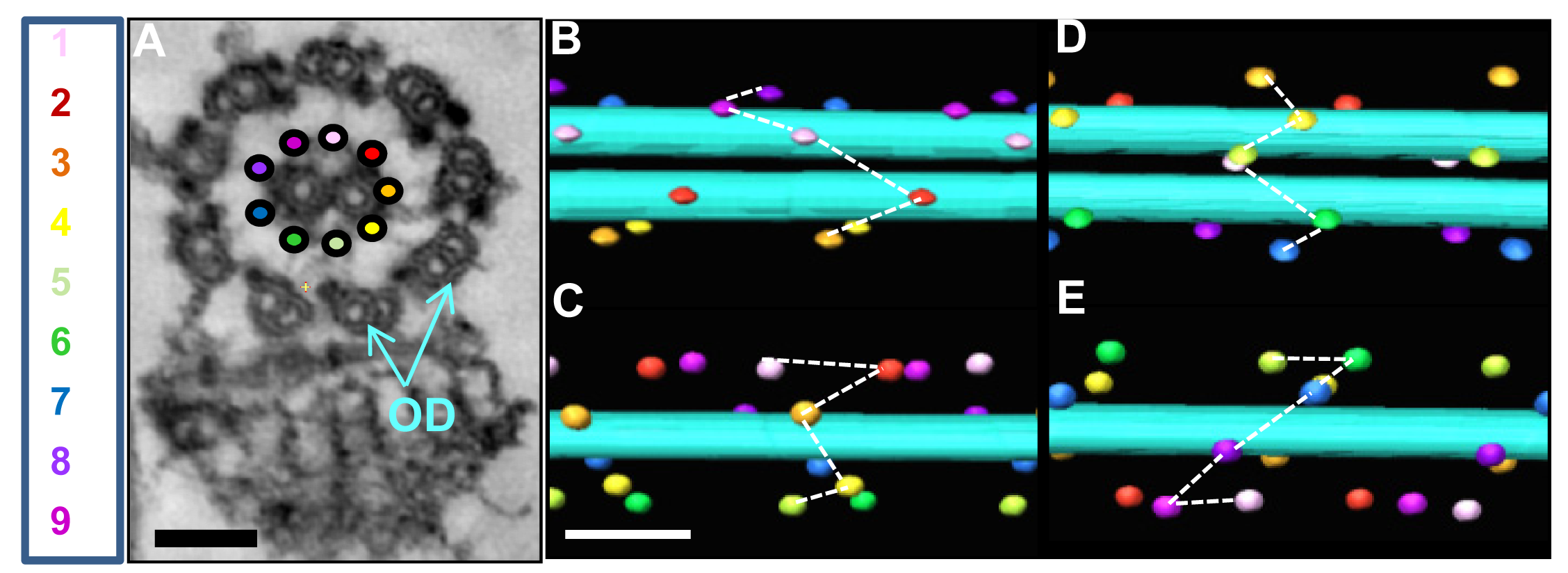

Supplement: Figure S2 — Bihelical arrangement of spoke triplets around the central pair microtubules. (A) Cross-sectional tomogram slice (≈50nm thick) shows a transverse view of the flagellum skeleton. Color scheme for spoke heads is based on outer doublet number as shown on the left, with outer doublet numbering as shown in figure 1. (B–E) Surface renderings of segmented tomograms show position of spoke heads around the central pair microtubules (light blue). Sample is oriented with flagellum base at left. Only the #1 spoke head from each triplet is shown, colored according to panel A. Each image is a 90° rotation toward the viewer of the previous image (B–E). White lines tracing the shortest distance to the spoke on the adjacent doublet moving 1 to 9, yield two hemi-helices of alternate handedness. Scale bars are 50nm. (TIF) [file pone.0025700.s002.tif]

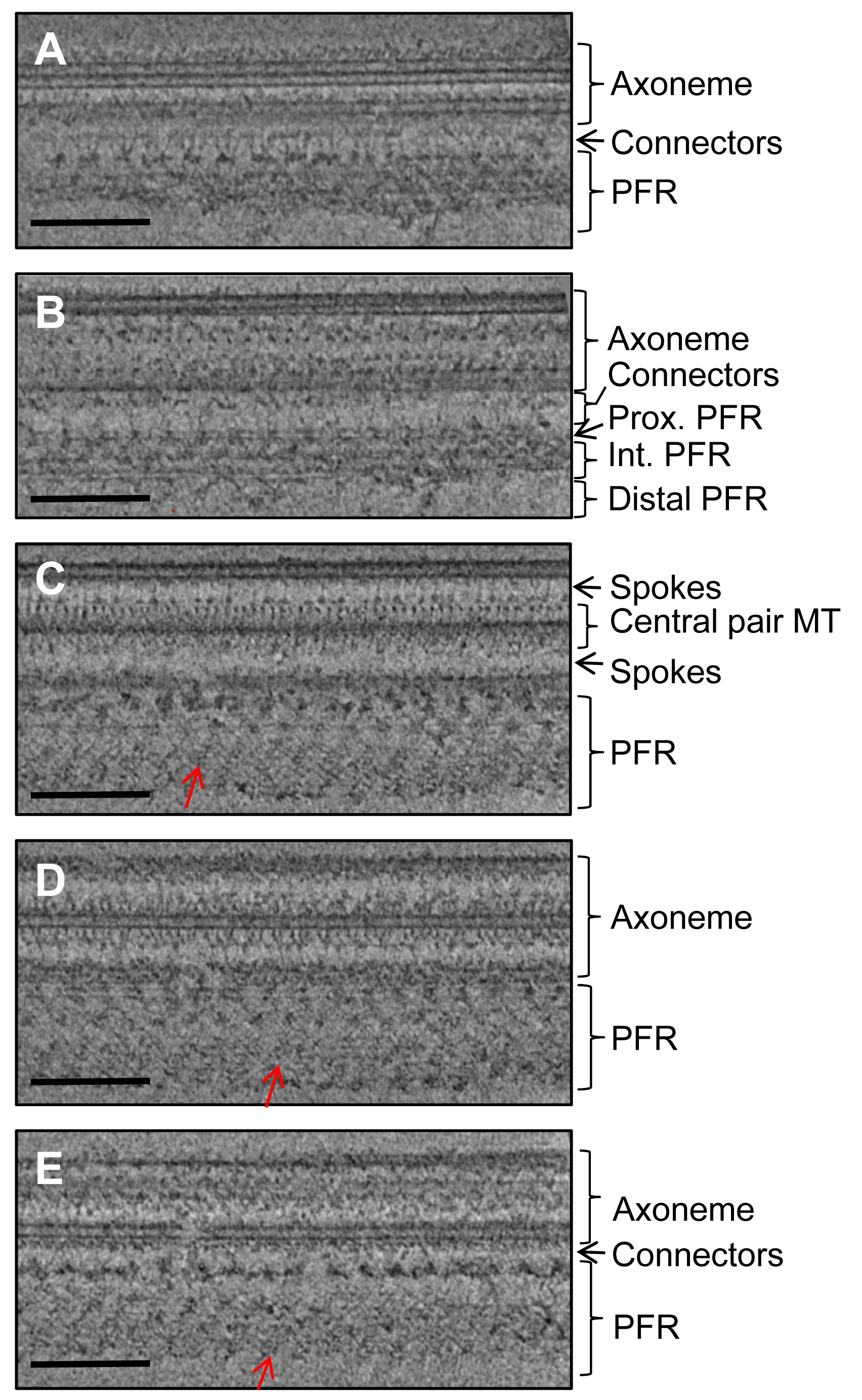

Supplement: Figure S3 — Serial tomogram slices of a frozen flagella skeleton showing the axoneme and PFR repeat. (A–E) Longitudinal section tomogram slices (≈1nm thick) show the axoneme and PFR repeat. (A) OD7-PFR connectors are in sync with periodic PFR densities. (B) OD6-PFR connectors are attached to periodic PFR densities in the proximal zone (Prox. PFR). Longitudinal fibrils are present in the intermediate (Int. PFR) and distal (Distal PFR) PFR zones. (C) Spoke triplets are arranged along the axoneme between the central pair microtubules and the outer doublets. (C–E) Distinct bands of periodic densities can be observed in the PFR (arrows). Scale bars are 100nm. (TIF) [file pone.0025700.s003.tif]

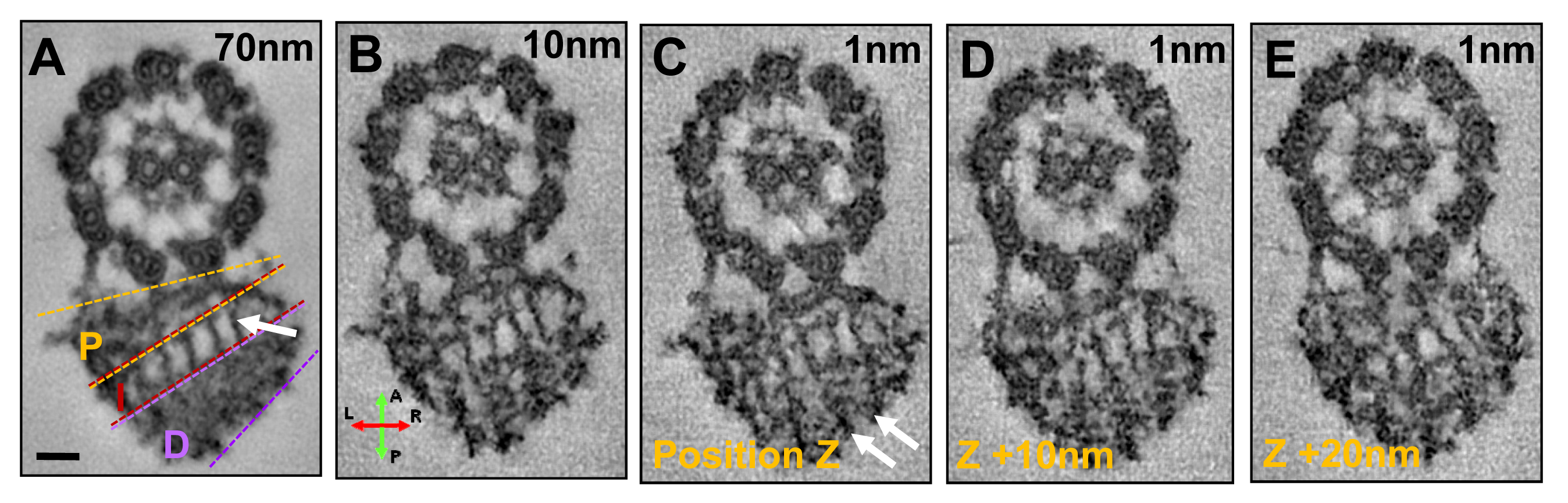

Supplement: Figure S4 — CS slices showing contiguous densities extending from proximal through to distal zones of the PFR. Transverse views of the flagellum skeleton. Slice thickness is indicated in each panel. The 70-nm thick slice shows the proximal (P), intermediate (I) and distal (D) PFR zones. Parallel linear densities are (white arrow) visible in the intermediate zone. Thin slices (10nm and 1nm) show densities extending across the PFR (white arrows). The x-y position of the proximal and distal segments of these contiguous densities displays variability in sequential thin z-slices. See also Video S2. Scale bar is 50nm. (TIF) [file pone.0025700.s004.tif]

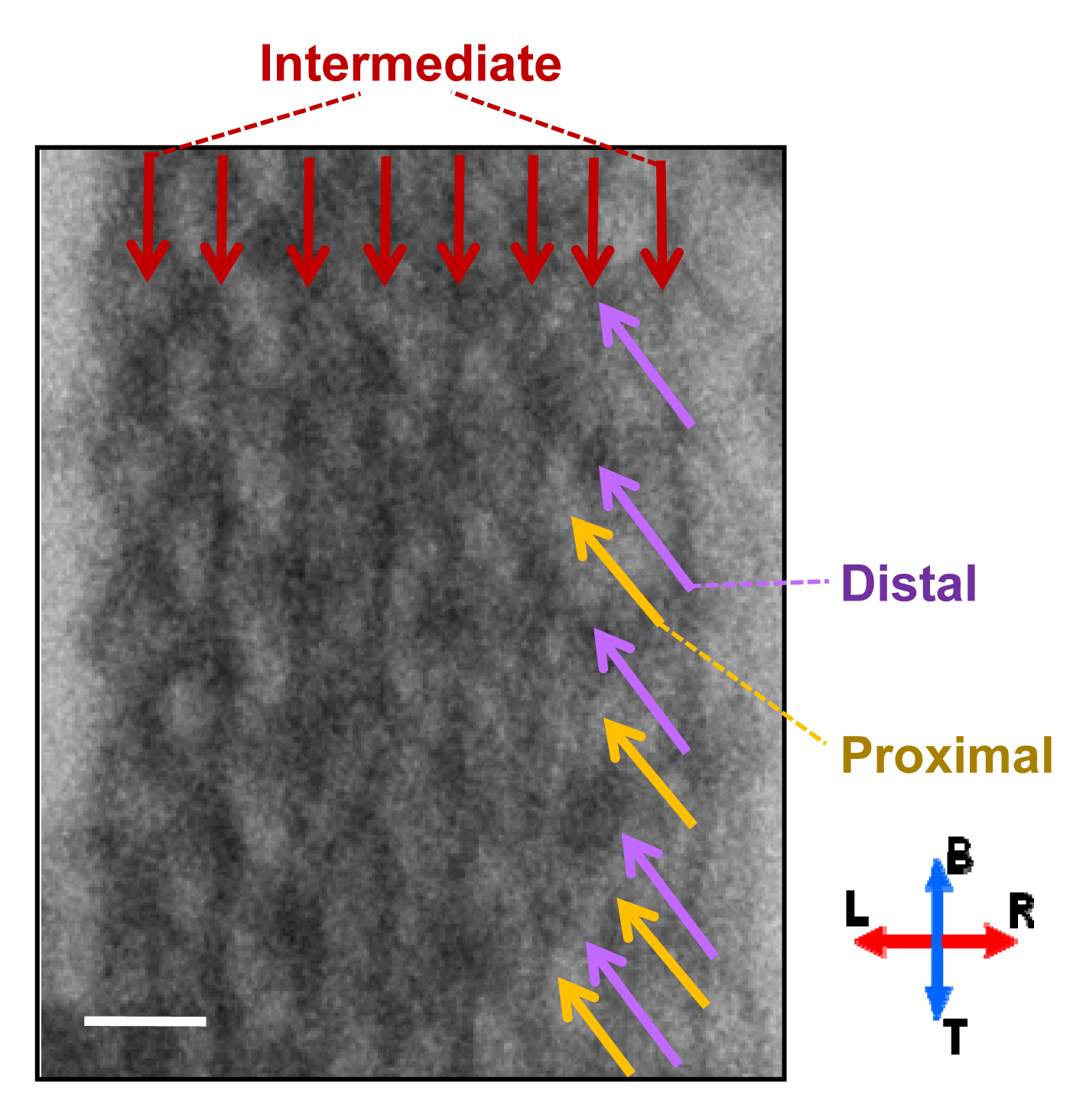

Supplement: Figure S5 — TEM image showing the relative positions of laths in each PFR zone. TEM image (∼200-nm thick) is of the same sample shown in 4C. Colored arrows indicate the position and direction of laths in the proximal (gold), intermediate (red) and distal (purple) zones. Proximal and distal laths are offset from one another by 24nm. Compass shows sample orientation, as described in Fig. 1. Scale bar is 50nm. (TIF) [file pone.0025700.s005.tif]

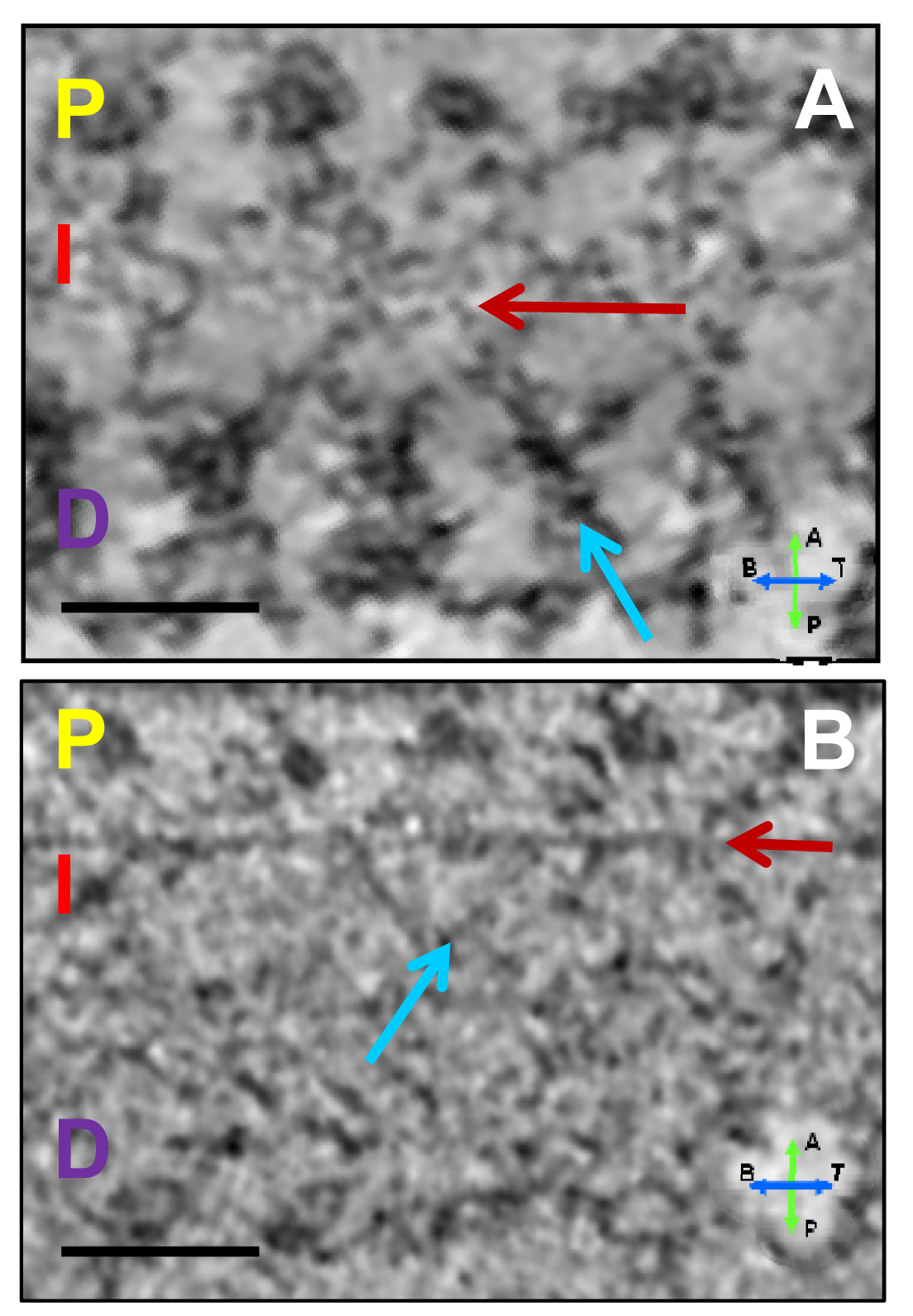

Supplement: Figure S6 — Digital slice from tomograms showing filaments in the PFR intermediate zone. (A, B) Sagittal tomogram slices (≈3nm thick) from a stained (A) and frozen (B) sample showing the 3 PFR zones, proximal (gold), Intermediate (red) and distal (purple). Filaments are observed in the intermediate zone parallel to the long axis of the flagellum (red arrows) and obliquely orientated, forming a connection from proximal through distal zones (blue arrows). Scale bar is 50nm. (TIF) [file pone.0025700.s006.tif]
